# Diet and prey selection of clouded leopards and tigers in Laos

**DOI:** 10.1002/ece3.9067

**Published:** 2022-07-05

**Authors:** Akchousanh Rasphone, Anita Bousa, Chantavy Vongkhamheng, Jan F. Kamler, Arlyne Johnson, David W. Macdonald

**Affiliations:** ^1^ Wildlife Conservation Society‐Lao PDR Program Vientiane Lao People's Democratic Republic; ^2^ Wildlife Conservation Research Unit The Recanati‐Kaplan Centre, Department of Zoology Oxford University Abingdon UK; ^3^ Anita Bousa, World Wide Fund for Nature—Laos Vientiane Lao People's Democratic Republic; ^4^ Chantavy Vongkhamheng, The Wildlife Conservation Association (Lao WCA) Vientiane Lao People's Democratic Republic; ^5^ Arlyne Johnson, Foundations of Success Bethesda Maryland USA

**Keywords:** Lao PDR, mainland serow, *Neofelis nebulosa*, niche breadth, *Panthera tigris*

## Abstract

In Southeast Asia, conservation of ‘Vulnerable’ clouded leopards (*Neofelis nebulosa*) and ‘Endangered’ tigers (*Panthera tigris*) might depend on the management of their preferred prey because large felid populations are limited by the availability of suitable prey. However, the diet of clouded leopards has never been determined, so the preferred prey of this felid remains unknown. The diet of tigers in the region has been studied only from one protected‐area complex in western Thailand, but prey preferences were not determined. To better understand the primary and preferred prey of threatened felids, we used DNA‐confirmed scats and prey surveys to determine the diet and prey selection of clouded leopards and tigers in a hilly evergreen forest in northern Laos. For clouded leopards, the primary prey was wild pig (*Sus scrofa*; 33% biomass consumed), followed by greater hog badger (*Arctonyx collaris*; 28%), small rodents (15%), and mainland serow (*Capricornis sumatraensis*; 13%; hereafter, serow). For tigers, the primary prey was wild pig (44%), followed by serow (18%), sambar (*Rusa unicolor*; 12%), and Asiatic black bear (*Ursus thibetanus*; 10%). Compared to availability, serow was positively selected by both clouded leopards (*D* = 0.69) and tigers (0.61), whereas all other ungulate species were consumed in proportion to the availability or avoided. Our results indicate that clouded leopards are generalist predators with a wide prey spectrum. Nonetheless, mid‐sized ungulates (50–150 kg) comprised nearly half of their diet, and were the preferred prey, supporting a previous hypothesis that the enlarged gape and elongated canines of clouded leopards are adaptations for killing large prey. Because serow was the only ungulate preferred by both felids, we recommend that serow populations be monitored and managed to help conservation efforts for clouded leopards and tigers, at least in hilly evergreen forests of Southeast Asia.

## Graphical abstract

We conducted the first ever dietary study of the mainland clouded leopard (Neofelis nebulosa), as well as the first dietary study of tigers (Panthera tigris) from hilly evergreen forests in Southeast Asia. Both felids consumed mostly wild pig (Sus scrofa), although mainland serow (Capricornis sumatraensis) was the only ungulate species that they preferred. Because populations of large felids are limited by availability of preferred prey, we recommend that populations of serow be monitored and managed to help conservation efforts for clouded leopards and tigers, at least in hilly evergreen forests of Southeast Asia.

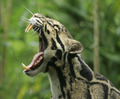

## 1 INTRODUCTION

Of the 10 large (>15 kg) felids worldwide, 9 of them have exhibited significant range declines and now are a conservation concern (Sandom et al., 2018). Prey loss is one of the primary reasons for large‐felid extinctions and range loss (Sandom et al., 2018), especially in the Indo‐Malayan region (Sandom et al., 2017). Because large felid populations are limited by prey availability, a major consideration of large felid conservation should be to maintain an adequate abundance of suitable prey that would be targeted by the felids (Clements et al., 2014; Karanth et al., 2004). Preferred prey (i.e., those targeted relative to abundance) are likely critical for tigers and other large carnivore populations because they provide the right‐sized food ‘packets’ that can be killed by females with relative safety and at a rate sufficient to raise cubs (Miller et al., 2014). Consequently, one of the main ecological drivers of tiger density across their range is the density of preferred prey (Miller et al., 2014). Overall, the carrying capacity of large carnivores can be better determined by the biomass of preferred prey, compared to total ungulate biomass (Hayward et al., 2007). Ultimately, determining preferred prey should be a priority for the conservation of large felids because management and enhancement of the preferred prey may be required for their persistence (Clements et al., 2014; Karanth et al., 2004).

In Southeast Asia, the numbers and distribution of mainland clouded leopards (*Neofelis nebulosa*) has declined dramatically during the last 30 years, resulting in fragmented and mostly isolated populations; consequently, this species is classified as ‘Vulnerable’ at the global level by the International Union for the Conservation of Nature (IUCN; Gray et al., 2021). The primary reasons for the decline are habitat loss and illegal hunting (D'Cruze & Macdonald, 2015), although prey depletion also might be a threat, especially in China and Indochina (Gray et al., 2021). Although conservation priority areas for clouded leopards have been determined in Southeast Asia based on their habitat preferences (Macdonald et al., 2019), no information exists regarding their preferred prey because dietary studies have not been conducted for this felid species.

**FIGURE 1:**
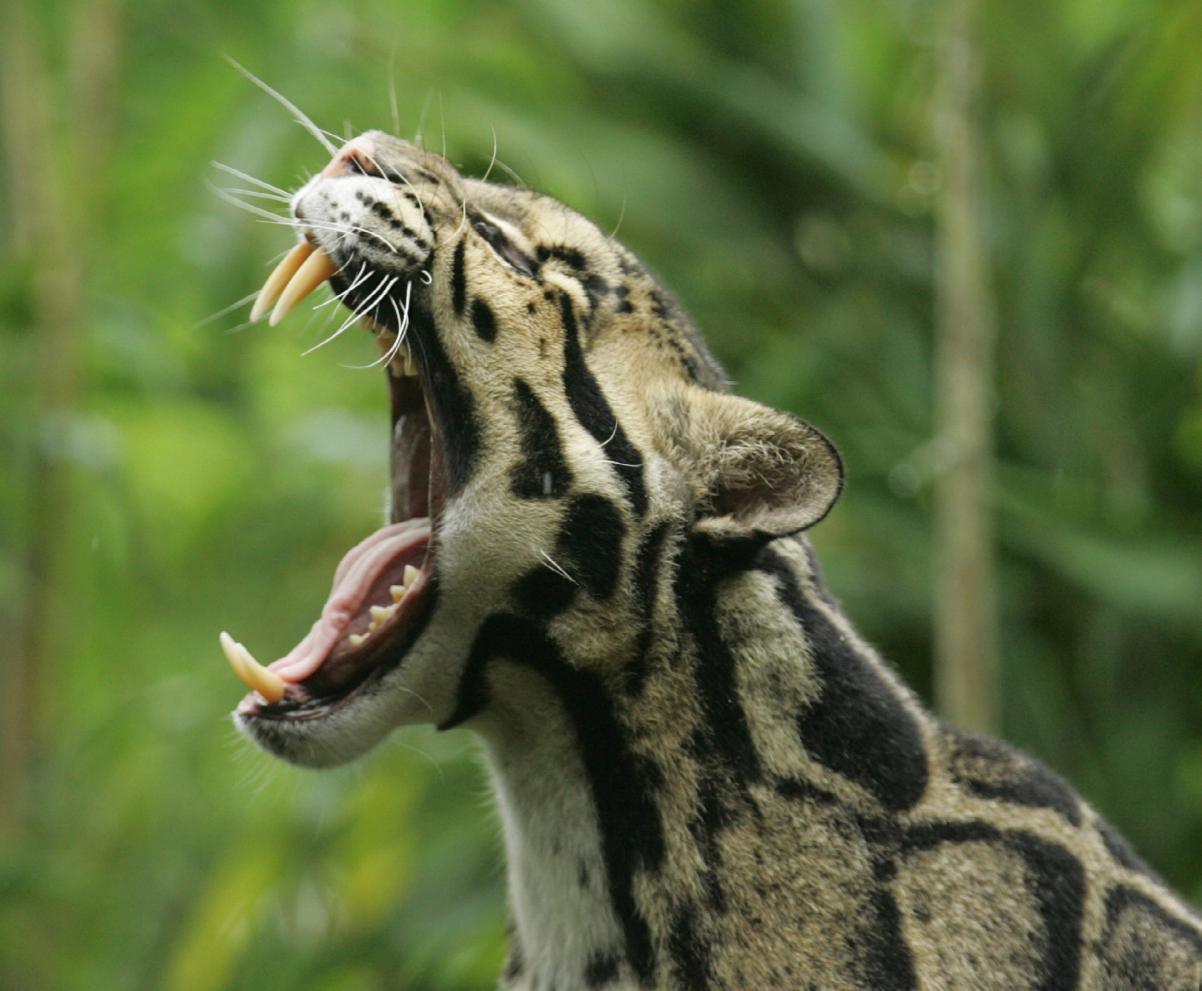
A yawning mainland clouded leopard demonstrates the longest relative canines and largest gape of any extant felid species (photograph by Christian Sperka Photography)

Clouded leopards are the smallest of the big cats (i.e., Pantherinae), but they have skull and dental morphologies that set them apart from all extant felids. They have the longest relative canines and largest gape of any extant felid species (Figure 1), and other dental, jaw, and skull morphologies that approach those of primitive saber‐toothed cats (Christiansen, 2006). These unique adaptations are probably related to major differences in killing behavior compared to other extant felids (Christiansen, 2006). Whereas most large felids subdue large prey with a suffocating throat bite, available evidence indicates clouded leopards can kill large prey with a powerful nape bite (Christiansen, 2006; Grassman Jr. et al., 2005; Rabinowitz et al., 1987). Pocock (1939) also speculated that due to their long canines and stocky build, clouded leopards seem best adapted to take large ungulate prey. Nevertheless, most observations concerning the predatory behavior of clouded leopards and their sister species, the Sunda clouded leopard (*Neofelis diardi*), seem to suggest that they mainly prey on primates and small ungulates such as muntjac (*Muntiacus* spp.; Matsuda et al., 2008; Morino, 2010; Nowell & Jackson, 1996; Sunderland‐Groves et al., 2021). In the most detailed description of clouded leopard diets to date, Grassman Jr. et al. (2005) found that clouded leopards in Thailand preyed on rodents, Sunda pangolin (*Manis javanica*), Bengal slow loris (*Nycticebus bengalensis*), and hog deer (*Axis porcinus*) based on 4 scats collected from captured clouded leopards and finding 2 presumed kills. More detailed information is needed about clouded leopard diets, especially for determining their preferred prey, which could aid conservation efforts given that prey depletion is a major threat for this species (Gray et al., 2021).

Tigers (*Panthera tigris*) are listed as ‘Endangered’ at the global level by the IUCN because of drastic reductions in their numbers and distribution across their range (Goodrich et al., 2015). The future of one of the least studied subspecies, the Indochinese tiger (*P. tigris corbetti*), is especially bleak because of recent extirpations in several countries and former source sites (Ash et al., 2020; O'Kelly et al., 2012; Rasphone et al., 2019). The Malayan tiger (*P. tigris jacksoni*), the only other tiger subspecies found in mainland Southeast Asia, also is experiencing a population crash and is heading toward extinction due to illegal killings and habitat loss (Ten et al., 2021).

The diet and prey selection of tigers has been determined from several sites in the Indian subcontinent and Russian Far East, and a review of dietary studies primarily from those regions showed that tigers preferentially preyed most on the wild pig (*Sus scrofa*) and sambar (*Rusa unicolor*; Hayward et al., 2012). However, the food habits of tigers in Southeast Asia are almost unknown. Dietary studies of the Indochinese tiger were only conducted in Thung Yai‐Huai Kha Khaeng (TY‐HKK) protected‐area complex in western Thailand. The TY‐HKK contains a seasonally dry habitat dominated by deciduous and mixed‐deciduous forests, and 4 dietary studies there gave conflicting results. Two studies found tigers consumed mostly large (>150 kg) ungulates, including banteng (*Bos javanicus*), gaur (*B. gaurus*), and sambar (Pakpien et al., 2017; Simcharoen et al., 2018), whereas the other 2 studies found that tigers consumed mostly smaller ungulates, such as wild pig and northern red muntjac (*Muntiacus vaginalis*; 20–28 kg; Prommakul, 2003; Rabinowitz, 1989). The diet of Malayan tigers has not been studied, and what little is known comes from a few scats and presumed kills of tigers from Taman Negara National Park (TNNP), Peninsular Malaysia, which showed that tigers preyed on sun bear (*Helarctos malayanus*; *n* = 3), domestic cattle (*Bos taurus*; *n* = 1), and Sunda pangolin (*n* = 1; Kawanishi & Sunquist, 2004). In contrast to TY‐HKK, the TNNP contains hilly evergreen forests where large ungulate (>150 kg) densities are relatively low compared to that of smaller ungulates (Kawanishi & Sunquist, 2004). More information about tiger diets is needed from Southeast Asia to aid conservation efforts there, given that prey numbers limit tiger population size (Karanth et al., 2004), and prey depletion is a major threat to tigers (Goodrich et al., 2015). Obtaining dietary data from closed evergreen forests are especially important because, in contrast to most other areas, large ungulates might not be the main prey of tigers in this habitat type (Sunquist et al., 1991).

We used DNA‐confirmed scats and prey surveys to determine the diet and prey selection of clouded leopards and tigers in a national park containing hilly evergreen forests in northern Lao People's Democratic Republic (hereafter, Laos). We predicted that clouded leopards would consume mostly primates and preferentially prey on muntjac, a common small ungulate found on the site (Johnson et al., 2006; Rasphone et al., 2019). Because large cervids and large bovids were relatively rare at this site (Johnson et al., 2006; Rasphone et al., 2019), we predicted that tigers would consume mostly wild pig, and that tigers would preferentially prey on wild pig and sambar, similar to that reported for tigers in other regions (Hayward et al., 2012).

## 2 METHODS

### 2.1 Study area

**FIGURE 2:**
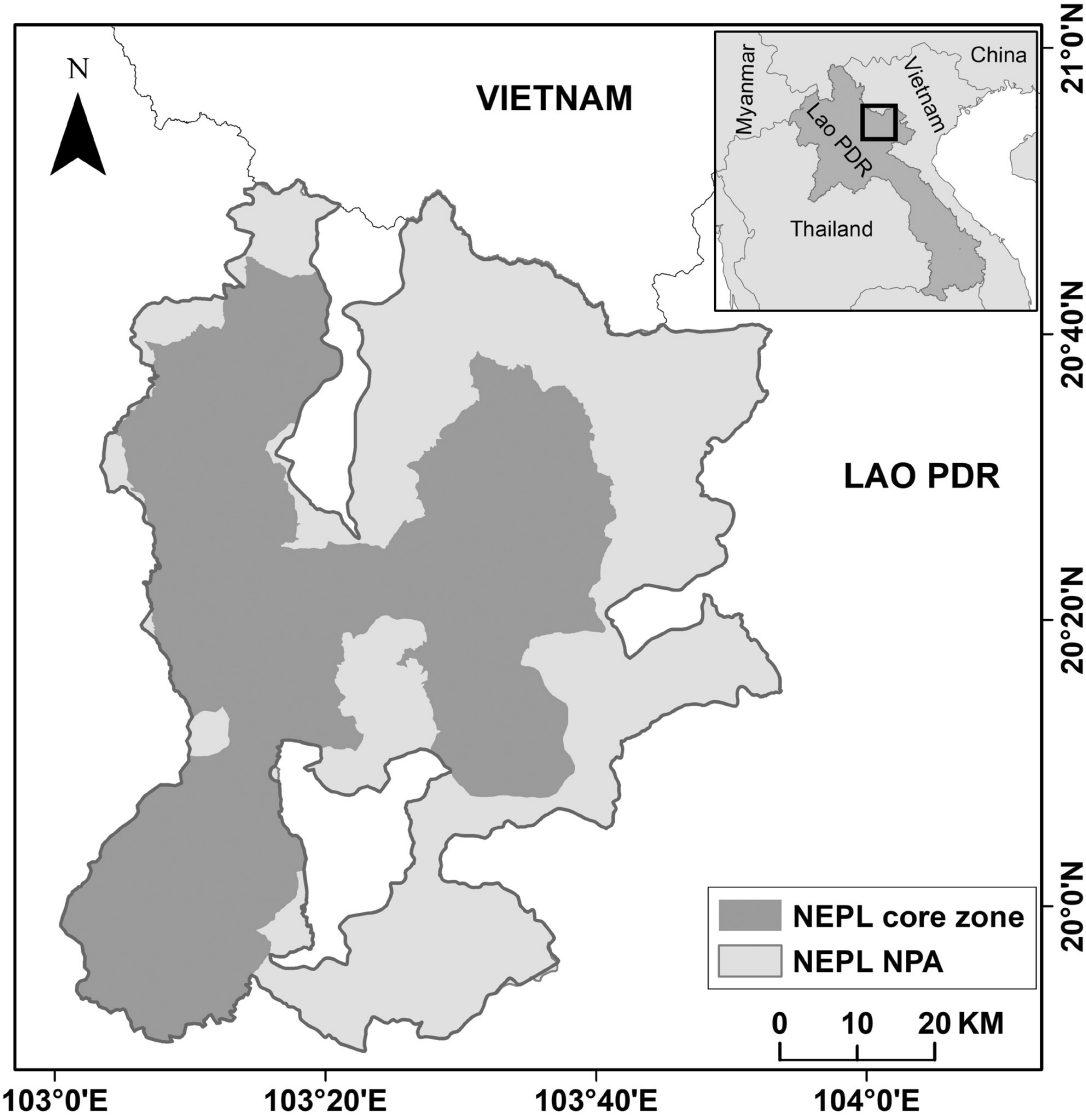
Location of Nam Et‐Phou Louey National Protected Area in northern Laos

We conducted research in the Nam Et‐Phou Louey National Protected Area (hereafter, NEPL) in northern Laos (5950 km^2^, Figure 2). Elevation in NEPL ranges from 400 to 2288 m, and it contains rugged and steep terrain with 91% of the area having slopes >12%. The vegetation is dominated by mixed evergreen‐deciduous forest up to 1500 m, transitioning into evergreen forest at 1500–1800 m, with interspersion of beech (*Fagus* spp.) and rhododendrons (*Rhododendron* spp.) >1800 m (Davidson, 1998). Annual rainfall is 1400–1800 mm, and temperatures range from 5°C (December–February) to 30°C (April–July). There are two main seasons: the rainy season (about 15 May to 31 October) and dry season (about 1 November to 14 May; Kamler, Thatdokkham, et al., 2020). The NEPL is known for its high biodiversity, especially carnivores and their prey (Johnson et al., 2006; Rasphone et al., 2019). Other felid species recorded in NEPL during the study include the Asian golden cat (*Catopuma temminckii*), leopard cat (*Prionailurus bengalensis*), and marbled cat (*Pardofelis marmorata*; Rasphone et al., 2019). Additional carnivore species include the dhole (*Cuon alpinus*), Asiatic black bear (*Ursus thibetanus*), and sun bear, as well as at least 13 small carnivore species (Rasphone et al., 2019). Prey species include wild ungulates such as gaur, sambar, mainland serow (*Capricornis sumatraensis*; hereafter, serow), wild pig, and at least two species of muntjac (primarily northern red muntjac), as well as 5 species of primates (Rasphone et al., 2019). The NEPL is officially divided into two zones: a totally protected core zone (3000 km^2^) where human activity (except for research and park management) is prohibited, and a peripheral managed‐use zone (2950 km^2^) where villages occur and specified livelihood activities (e.g., collection of non‐forest timber products, subsistence hunting of common species with traditional weapons) are permitted following park regulations (Figure 1). All of our research activities were carried out in the core zone.

### 2.2 Scat analysis and prey selection

Diets of clouded leopards and tigers were determined by analysis of scats (i.e., feces) that were opportunistically collected on trails by researchers and park staff in NEPL from January 2008 to March 2012. Most scats were collected while conducting camera‐trap surveys and grid‐based occupancy surveys that covered the entire core zone (Johnson et al., 2016; Vongkhamheng et al., 2013); therefore, we assumed the collected scats represented a random sample of the felid populations. For each scat, the scat diameter (when possible), date, and GPS location were recorded, and then scats were stored in plastic bags with silica pouches to desiccate them. Up to 10 g of each scat were sent to the Sackler Institute for Comparative Genetics, American Museum of Natural History (New York) for species identification based on mitochondrial DNA analysis (see Caragiulo et al., 2014 for methodological details). Remaining parts of the scats were washed over a sieve, dried for several days, and then contents were separated. Hair samples from each scat were identified to species by examining the structures of the cuticle, medulla, and cross sections under a microscope, and comparing those to a reference collection of hairs from known species. Our data collection did not involve direct handling of study animals; therefore, our research was exempt from review by the University of Oxford, Biomedical Sciences, Animal Welfare and Ethical Review Body (AWERB).

Results from scat analysis were quantified in terms of the percent biomass consumed because this method provides the most accurate estimate of carnivore diets using correction factors that account for differential digestibility of food items (Klare et al., 2011). Following the recommendations by Klare et al. (2011), we also included percent volume of food items, and the frequency of occurrence (i.e., percentage of scats containing a particular food item) to make our results more comparable to previous studies. To estimate biomass consumed, we followed Chakrabarti et al. (2016), who developed a generalized model (biomass consumed per collectable scat/predator weight = 0.033–0.025exp^−4.284[weight of prey killed/predator weight]^) based on feeding trials of several felid species ranging in size from the domestic cat (*Felis silvestris catus*) to the lion (*Panthera leo*). To determine if we obtained the minimum number of scats needed to adequately describe the diets of clouded leopards and tigers, we calculated a prey‐species accumulation curve using the ‘Vegan’ package v. 2.5–6 with the function *specaccum* (Jaimes et al., 2018; Oksanen et al., 2019) in R version 4.0.5 (R Core Team, 2021). We calculated the expected mean prey richness and standard deviation with 10,000 permutations to obtain 95% confidence intervals (Gotelli & Colwell, 2001).

For the biomass consumed models (Chakrabarti et al., 2016), we used 17 kg for clouded leopards and 172.5 kg for tigers, which were the median of the weight ranges given by Nowell and Jackson (1996) and Francis (2008). For the prey weights of clouded leopards, we used half the weight of adult females for wild pig (37.5 kg) and serow (55 kg; Francis, 2008) because we assumed clouded leopards killed individuals ranging in size from young to adult females. We used a weight of 10.5 kg for the greater hog badger (*Arctonyx collaris*, hereafter hog badger; Francis, 2008), 2.75 kg for the Asiatic brush‐tailed porcupine (*Atherurus macrourus*; Nowak, 1999), and 0.5 kg for rats (i.e., large murids). Because civet hairs often could not be identified to species, we used the mean of the adult weights of the large Indian civet (*Viverra zibetha*; 8.5 kg), common palm civet (*Paradoxurus hermaphroditus*; 2.5 kg), and masked palm civet (*Paguma larvata*; 4 kg), as these were the 3 most common civet species on the study site based on the camera trap data (Rasphone et al., 2019). We used a live weight of 0.5 kg for birds, assuming that clouded leopards consumed birds ranging in size from large songbirds (ca. 100 g) to red junglefowl (*Gallus gallus*; 1 kg). For the prey weights of tigers, we used 38 kg for wild pig and 212 kg for sambar, which was the mean weight of individuals killed by tigers in India (Karanth & Sunquist, 1995). We used the adult weights of serow (135 kg), muntjac (24 kg) and stump‐tailed macaque (*Macaca arctoides*; 10 kg; Francis, 2008), and the half the adult female weight for Asiatic black bears (50 kg), assuming tigers killed bears ranging in size from young to adult females. The weights for hog badgers and civets were the same as those used for clouded leopards. Based on the biomass of prey categories consumed, we calculated Levin's measure of niche breadth (*B*; Krebs, 1989) for each felid species.

To determine prey selection of ungulate species consumed by clouded leopards and tigers, we calculated Jacobs' (1974) electivity index *D* based on biomass consumed versus biomass available. To determine biomass available for each ungulate species, we multiplied adult female weights (i.e., weight of an average‐sized individual within the population) by estimates of ungulate densities on our site. Ungulate densities were estimated in the core zone of NEPL in 2008 using a grid‐based occupancy survey (Vongkhamheng et al., 2013), and results (individuals ± SE/km^2^) were: muntjac, 1.50 ± 0.11; wild pig, 3.19 ± 0.15; sambar, 0.36 ± 0.01; serow, 0.22 ± 0.02; and gaur, 0.02 ± 0.003. For the available biomass calculations, we used adult female weights of 20 kg for red muntjac, 75 kg for wild pig, 85 kg for serow, and 185 kg for sambar, which were based on lower weight given for each species by Francis (2008). Because *D*‐values of rare species often are biased, we used only those species that were >5% of the biomass available (Klare et al., 2010); thus, *D*‐values were not calculated for gaur. Following Laurenzi et al. (2016) who used the similar Ivlev's electivity index, we considered *D*‐values to show preference if values were >0.30, and avoidance if values were <−0.30.

## 3 RESULTS

**FIGURE A1:**
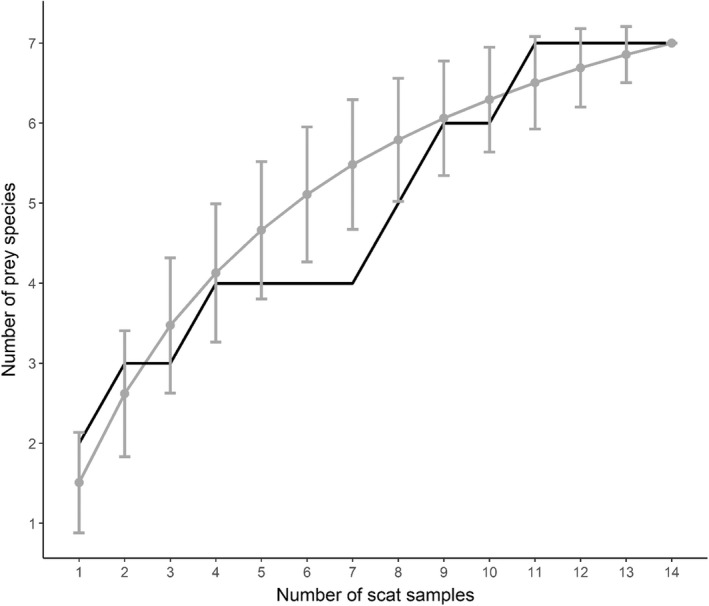
Accumulation curve (gray line) of expected mean prey richness from clouded leopard scats collected in Nam Et‐Phou Louey National Protected Area, Laos, 2008–2012. The bars represent 95% confidence intervals based on 10,000 permutations. The black line represents the actual accumulation of prey species based on scat content in the order in which scats were found

There were 14 scats of clouded leopards and 21 scats of tigers that were confirmed by DNA analysis to be from those species, out of 361 scats collected. Mean (±SD) scat diameter was 2.1 ± 0.3 cm (range = 1.5–2.5 cm) for clouded leopards, and 4.0 ± 0.6 cm (range = 3.5–5.5 cm) for tigers. Most scats of clouded leopards (11/14) and tigers (17/21) were collected during the dry season. For clouded leopards, a majority of scats (8/14) contained 1 prey item, whereas remaining scats contained 2 or 3 prey items. Overall, we identified 7 prey species in their scats, including 2 ungulate species and at least 2 carnivore species (Table 1). Ungulates comprised 46% of all biomass consumed, followed by carnivores (33%) and rodents (18%, Table 1). Wild pig was the most dominant prey item (33% of biomass consumed), followed by hog badger (28%), small rodent (15%), and serow (13%), whereas no other species was >10% (Table 1). The prey accumulation curve for clouded leopards showed that an asymptote was not yet reached for the simulated data, although an asymptote was reached at 11 scats for the actual data (Figure A1).

**TABLE 1:** Diet composition expressed as percentage of ingested biomass (Bio), percentage of scat volume (Vol), frequency of occurrence per scat (Occ), and dietary niche breadth (*B*) of clouded leopards and tigers in Nam Et‐Phou Louey National Protected Area, Laos, 2008–2012

	Clouded leopard (*n* = 14 scats)			Tiger (*n* = 21 scats)		
Prey category	Bio	Vol	Occ	Bio	Vol	Occ
Ungulate	46.1	33.3	35.7	81.1	74.6	85.7
Wild pig (*Sus scrofa*)	33.3	23.8	28.6	43.8	44.0	52.4
Mainland serow (*Capricornis sumatraensis*)	13.3	9.5	14.3	18.0	13.1	19.0
Sambar (*Rusa unicolor*)	0.0	0.0	0.0	12.2	8.7	14.3
Muntjac (*Muntiacus* spp.)	0.0	0.0	0.0	7.2	8.7	14.3
Carnivore	32.7	25.0	28.6	17.5	23.0	33.3
Greater hog badger (*Arctonyx collaris*)	28.4	21.4	28.6	5.6	9.5	14.3
Civet^a^	4.2	3.6	7.1	2.4	4.8	4.8
Asiatic black bear (*Ursus thibetanus*)	0.0	0.0	0.0	9.6	8.7	14.3
Macaque (*Macaca* spp.)	0.0	0.0	0.0	1.4	2.4	4.8
Rodent	18.1	35.2	50.0	0.0	0.0	0.0
Brush‐tailed porcupine (*Atherurus macrourus*)	3.1	3.6	7.1	0.0	0.0	0.0
Small rodent (Muridae)	14.7	31.6	50.0	0.0	0.0	0.0
Bird	3.0	6.4	14.3	0.0	0.0	0.0
Niche Breadth *B*	4.27			3.89		

^a^ Probably the common palm civet (*Paradoxurus hermaphroditus*), masked palm civet (*Paguma larvata*), and/or large Indian civet (*Viverra zibetha*).

**FIGURE A2:**
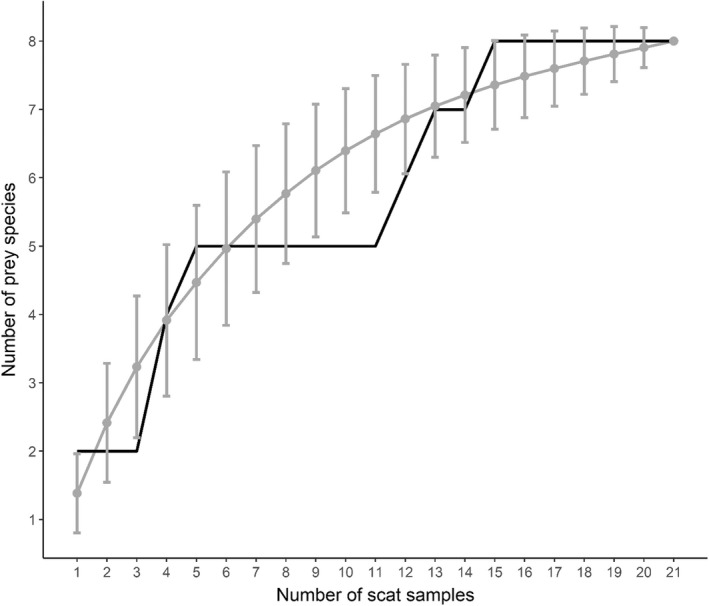
Accumulation curve (gray line) of expected mean prey richness from tiger scats collected in Nam Et‐Phou Louey National Protected Area, Laos, 2008–2012. The bars represent 95% confidence intervals based on 10,000 permutations. The black line represents the actual accumulation of prey species based on scat content in the order in which scats were found

For tigers, a majority of scats (14/21) contained 1 prey item, whereas remaining scats contained 2 or 3 prey items. Overall, we identified 8 prey species in their scats, including 4 ungulate species and at least 3 carnivore species (Table 1). Ungulates comprised 81% of all biomass consumed, with carnivores being the only other significant prey group consumed (18%; Table 1). Wild pig was the most dominant prey item (44% of biomass consumed), followed by serow (18%), sambar (12%), and Asian black bear (10%), whereas no other species was ≥10% (Table 1). The prey accumulation curve for tigers approached an asymptote after 19 scats for the simulated data (based on upper limit of 95% CI's), and an asymptote was reached at 15 scats for the actual data (Figure A2).

**FIGURE 3:**
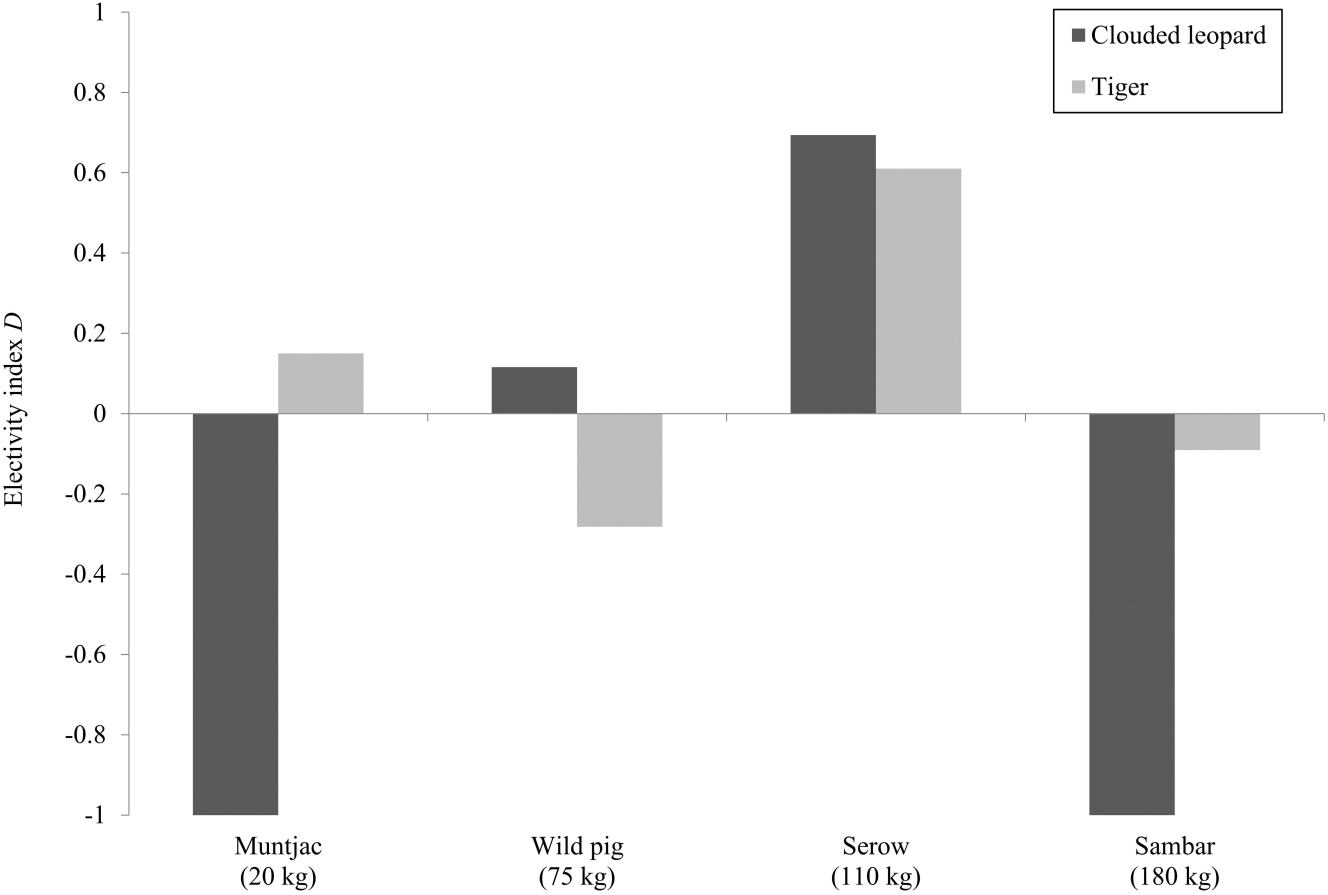
Jacobs' (1974) electivity index (*D*) of ungulates based on percent biomass consumed by clouded leopards and tigers in Nam Et‐Phou Louey National Protected Area, Laos, 2008–2012. Body mass of each species represents the adult female body mass reported by Francis (2008)

The biomass of ungulates consumed by clouded leopards and tigers did not reflect the biomass available because both felids showed a preference for serow (*D* = 0.69 and 0.61, respectively; Figure 3). Clouded leopards showed no selection for wild pig (0.12), and a strong avoidance of both muntjac and sambar (−1.0 for both; Figure 23). Tigers showed no selection for muntjac (0.01), sambar (−0.09), and wild pig (*D* = −0.28, Figure 3). Overall, niche breadth values (*B*) were slightly higher for clouded leopards (4.27) compared to tigers (3.89, Table 1).

## 4 DISCUSSION

The diet of clouded leopards did not include primates or muntjac, which did not support our prediction. The lack of primates in our sample of the clouded leopard scats was surprising, given that previous studies assumed that clouded leopards and Sunda clouded leopards were major predators of primates (Matsuda et al., 2008; Morino, 2010; Nowell & Jackson, 1996; Sunderland‐Groves et al., 2021). Although we did not determine primate densities on our study site, four macaque species were present on our study site (Rasphone et al., 2019), and overall macaques were relatively abundant based on the camera‐trap data (Johnson et al., 2006; Rasphone et al., 2019). Perhaps predation on primates was low in NEPL because macaques exhibit group defense and have formidable defense weaponry (i.e., large canines) to deter predation. Muntjac had the second highest density of all ungulates in NEPL (Vongkhamheng et al., 2013), yet they were not detected in clouded leopard scats. This was surprising given that concurrent studies showed that muntjac were 49% of the dhole diet and 22% of the Asian golden cat diet in NEPL (Kamler, Inthapanya, et al., 2020; Kamler, Thatdokkham, et al., 2020). Clouded leopards are similar in size to muntjac, so we assumed that if they predated on ungulates, they would focus on muntjac similar to that shown for the other mid‐sized carnivores, but this was not supported by our results.

Instead of relatively small prey, nearly half of the clouded leopard diet was comprised of medium‐sized (50–150 kg) ungulates. Overall, wild pig was the primary prey of clouded leopards, whereas serow was the only ungulate preferred based on availability. Preference for serow by clouded leopards was unique among the other sympatric carnivores because concurrent dietary studies showed that neither dholes nor Asian golden cats preferentially consumed serow (Kamler et al., 2012; Kamler, Inthapanya, et al., 2020; Kamler, Thatdokkham, et al., 2020). Serow might even have influenced the activity patterns of clouded leopards, because a recent study in NEPL showed that clouded leopard had high activity overlap with serow (Δ = 0.75), compared to lower activity overlap with northern red muntjac (0.65), squirrels (0.63), wild pig (0.60), birds (0.53), and 4 macaque species (0.41–0.52; Rasphone et al., 2020). Previous research has shown that felids often synchronize their activity to that of their main prey (Foster et al., 2013; Nagy‐Reis et al., 2019; Yang et al., 2018). Interestingly, clouded leopards weigh 11–23 kg (Francis, 2008; Nowell & Jackson, 1996), so they overlap the 21.5–25 kg weight range where carnivores shift from small prey (less than half of carnivore mass) to large prey (near or greater than predator mass; Carbone et al., 1999), indicating clouded leopards taking large prey is consistent with carnivore energetic models. Although we could not determine if clouded leopards were feeding on adult or young ungulates, they mostly consumed medium‐sized ungulate species when more abundant and smaller ungulate species were available is significant. Serow were not likely scavenged by clouded leopards because concurrent dietary studies in NEPL showed that serow was consumed in minimal amounts by dholes (6% of diet; Kamler, Thatdokkham, et al., 2020), Asian golden cats (1%; Kamler, Inthapanya, et al., 2020), and leopard cats (0%; Kamler, Inthapanya, et al., 2020), suggesting that serow carcasses were not readily available to carnivores on the study site. Overall, our results support the hypothesis by Pocock (1939) and Christiansen (2006) that the elongated canines, large gape, and stocky build of the clouded leopard are adaptations to regularly take prey much larger than their own body size.

Hog badgers were the second most important prey item of clouded leopards, and overall carnivores comprised nearly one third of the clouded leopard diet, which was unexpected. Regarding other carnivores as prey, there was one previous report of a clouded leopard preying on a binturong (*Arctictis binturong*; Lam et al., 2014), and one previous report of a Sunda clouded leopard preying on a common palm civet (Rabinowitz et al., 1987). Greater hog badgers (7–14 kg; Francis, 2008) overlap in weight with clouded leopards, so it was somewhat surprising that clouded leopards fed more on this carnivore species compared to smaller carnivores species, such as the 4 civet species which are relatively abundant and widespread in NEPL (Rasphone et al., 2019). Perhaps their elongated canines and stocky build allows clouded leopards to regularly predate on similarly sized carnivores, which, based on optimal foraging theory, would be more energetically profitable than feeding on smaller carnivores, assuming that handling time is similar (MacCracken & Hansen, 1987). Hog badgers were not likely scavenged by clouded leopards because concurrent dietary studies in NEPL showed that hog badgers were consumed in minimal amounts by dholes (3% of diet; Kamler, Thatdokkham, et al., 2020), Asian golden cats (4%; Kamler, Inthapanya, et al., 2020), and leopard cats (0%; Kamler, Inthapanya, et al., 2020), suggesting that hog badger carcasses were not readily available to carnivores on the study site.

Small rodents were frequently consumed by clouded leopards in NEPL, as they were found in 50% of all clouded leopard scats. Nonetheless, small rodents contributed less to the biomass consumed by clouded leopards compared to ungulates and other carnivores. Small rodents reportedly were consumed by clouded leopards in Thailand (Grassman et al., 2005), so clearly small prey are regularly consumed by clouded leopards despite their apparent adaptations for killing large prey. Because clouded leopards overlap the 21.5–25 kg weight range that signals a major shift in carnivore diets, this species can energetically sustain itself on both large and small prey, unlike larger felids that are more energetically restricted to large prey (Carbone et al., 1999; Miller et al., 2014). Perhaps by having a body size that is capable of consuming both large and small prey, clouded leopards can coexist within diverse felid communities because they are able to reduce prey competition with large apex felids as well as small felids. Our results support this hypothesis because the clouded leopard had a relatively high dietary niche breadth that included a wide spectrum of prey sizes ranging from 0.5 kg to >100 kg.

Ungulates comprised 81% of the tiger diet, and wild pig were the most common prey item, which supported our prediction. In fact, wild pig was a higher component of the tiger diet in NEPL (34%) than in previous studies in TY‐HKK, Thailand, where wild pig comprised 2–11% of the tiger diet (Pakpien et al., 2017; Prommakul, 2003; Rabinowitz, 1989; Simcharoen et al., 2018). Higher consumption of wild pig in NEPL probably was related to the scarcity of large bovids and sambar, as the latter two typically were the main prey of tiger in TY‐HKK (Pakpien et al., 2017; Prommakul, 2003; Simcharoen et al., 2018). Despite the high consumption, wild pig was not preferred prey of tigers in NEPL, which did not support our prediction. Similarly, sambar was consumed by tigers in NEPL, but it was not preferred prey, which also did not support our prediction. Our results are in contrast to other regions where wild pig and sambar were found to be the most preferred prey of tigers (Hayward et al., 2012).

Unexpectedly, serow was the second most important prey of tigers in NEPL, and the only preferred ungulate prey. To our knowledge, only one previous study reported that tigers consumed serow; in TY‐HKK serow comprised 1 of 150 tiger kills (Pakpien et al., 2017). The body mass of serow (110–160 kg; Francis, 2008) is within the preferred prey weight range of tigers (60–250 kg; Hayward et al., 2012); thus, it was not surprising that tigers selectively consumed an ungulate species of this size. However, it was surprising that tigers selected serow instead of wild pig and sambar, because the latter two species also are within the preferred weight range of tigers and both are considered the tiger's most preferred prey in Russia and the Indian subcontinent (Hayward et al., 2012). Serow are rare or absent in Russia and the Indian subcontinent, whereas in Southeast Asia this species is somewhat widespread, especially in closed evergreen forests with rugged terrain (Phan et al., 2020) such as NEPL. When all three ungulate species are sympatric, we speculate that tigers prefer serow because this is species is solitary and has less defense weaponry compared to wild pig and sambar; thus, serow potentially could be easier to stalk and safer to handle compared to the group‐living wild pig and sambar. Also, because serow use more rugged and steep terrain compared to sambar and wild pig, then serow might have been more accessible to tigers if the latter used a hunting strategy that took advantage of this terrain. Prey accessibility has been shown to be more important than prey numbers regarding habitat selection and hunting strategy of large felids (Balme et al., 2007; Rostro‐García et al., 2015).

Carnivores comprised 17% of the tiger diet in NEPL, mainly Asiatic black bears but also hog badgers and civets. Previous reviews found that tigers regularly consumed bears in the Russian Far East (Seryodkin et al., 2018), as well as other areas of Asia including Southeast Asia (Naing et al., 2019). Thus, it was not surprising to find bears in the tiger diet in NEPL, although at 10% of diet, bears appear to be more important prey to tigers in Southeast Asia than was previously assumed, at least in closed evergreen forests. Limited data from Peninsular Malaysia support this conclusion, because in the closed evergreen forests of Taman Negara National Park, bears comprised 3 of 5 tiger kills and scats that were found (Kawanishi & Sunquist, 2004).

In the core zone of NEPL, the estimated tiger population based on camera‐trap surveys in 2003–2004 was 7–23 individuals (Johnson et al., 2006). Based on DNA analysis of tiger scats collected from 2008 to 2010, there were a minimum of 16 different individuals (Vongkhamheng, 2011), suggesting the tiger population remained low but stable in the NEPL core zone during our study. However, because of an exponential increase in illegal snaring that began after 2010 (Johnson et al., 2016), the tiger population quickly decreased to a minimum of 2 individuals by 2013, and soon thereafter likely became extirpated (Rasphone et al., 2019). Nonetheless, most of our data were collected before the exponential increase in snaring, and we assume our results adequately represent tiger dietary habits in a low‐density population occupying hilly evergreen forests.

A major limitation of our study was the low sample of scats for each felid species, especially for clouded leopards because the simulated data for their prey species did not reach an asymptote. Therefore, the lack of primates and muntjac detected in clouded leopard scats could have been an artifact of our low sample size. Pseudo‐replication might affect small sample sizes from low density populations, although that was not likely the case in our study. For example, more detailed genetic analysis on the first 16 tiger scats collected found that they came from 16 different individuals (Vongkhamheng, 2011). For clouded leopards, a detailed camera‐trap study from 2013 to 2017 estimated a density of 1.8 individuals/100 km^2^ in our study site with a minimum of 41 different individuals detected and a high turnover of individuals between years (Rasphone et al., 2021); thus, the scats we collected over 4 years likely were not biased towards a few individuals. Interestingly, clouded leopards apparently do not defecate on trails to mark their territories (Rabinowitz et al., 1987), in contrast to other felids (Harmsen et al., 2010; Kamler, Inthapanya, et al., 2020; Rodgers et al., 2015), which may explain the low sample size in our study, and why no previous dietary studies were conducted on clouded leopards (i.e., researchers rarely, if ever, find scats of this species). Nevertheless, if male clouded leopards defecated on trails at a higher rate than females, our results could have been sex biased. Finally, because most scats from both felids were collected during the dry season, our results could have had a seasonal bias if predatory habits differed between the dry and rainy seasons. For example, ungulate birth pulses, rutting behavior, and migration might result in a seasonal variation in carnivore diets, including the tiger (Schaller, 1967). Nonetheless, a concurrent study of dhole diets in NEPL with a large sample size (*n* = 165 scats) showed there was no significant difference between the dry and rainy seasons (Kamler, Thatdokkham, et al., 2020), suggesting seasonal differences in diets of tigers and clouded leopards in NEPL could have been minimal.

### 4.1 Conservation implications

Our study represents the first ever dietary study of clouded leopards, and the first dietary study of tigers in Southeast Asia from hilly evergreen forests. Because serow was the only ungulate preferred by clouded leopards and tigers, this prey species likely is important for the conservation of both felids, at least in some areas of Southeast Asia. In protected areas containing hilly evergreen forests where clouded leopards or tigers occur, we recommend that park managers monitor serow populations to ensure that both large felids have an adequate population of their preferred prey. The mainland serow is classified as ‘Vulnerable’ at the global level by the IUCN because its populations are fragmented and in rapid decline due to poaching and habitat loss (Phan et al., 2020). Therefore, reintroductions or supplemental releases of serow might be necessary in areas where serow populations have become decimated by poaching because scarcity of preferred prey can hamper the recovery of tigers and other large felids (Clements et al., 2014; Hayward et al., 2007; Karanth et al., 2004; Miller et al., 2014; Sandom et al., 2017, 2018). We recommend further research on the diets of clouded leopards and tigers in Southeast Asia, including larger sample sizes from all seasons and data from sites with different habitat and prey communities. Only by studying these felids in various ecosystems can we better understand their predatory niches and determine their ecological requirements.

## AUTHOR CONTRIBUTIONS

**Akchousanh Rasphone:** Conceptualization (lead); formal analysis (lead); investigation (lead); methodology (lead); writing – original draft (lead). **Anita Bousa:** Methodology (equal); writing – review and editing (supporting). **Chantavy Vongkhamheng:** Methodology (equal); writing – review and editing (supporting). **Jan F Kamler:** Conceptualization (equal); formal analysis (equal); methodology (equal); writing – original draft (supporting). **Arlyne Johnson:** Funding acquisition (equal); project administration (supporting); writing – review and editing (supporting). **David Macdonald:** Funding acquisition (equal); writing – review and editing (supporting).

## CONFLICT OF INTEREST

The authors declare that they have no competing interests.

## Data Availability

Results of the analysis of individual scats are available via DRYAD: https://doi.org/10.5061/dryad.w0vt4b8v5.

## References

[ece39067-bib-0001] Ash, E. , Hallam, C. , Chanteap, P. , Kaszta, Ż. , Macdonald, D. W. , Rojanachinda, W. , Redford, T. , & Harihar, A. (2020). Estimating the density of a globally important tiger (*Panthera tigris*) population: Using simulations to evaluate survey design in eastern Thailand. Biological Conservation, 241, 108349.

[ece39067-bib-0002] Balme, G. , Hunter, L. , & Slotow, R. (2007). Feeding habitat selection by hunting leopards *Panthera pardus* in a woodland savanna: Prey catchability versus abundance. Animal Behaviour, 74, 589–598.

[ece39067-bib-0003] Caragiulo, A. , Dias‐Freedman, I. , Clark, J. A. , Rabinowitz, S. , & Amato, G. (2014). Mitochondrial DNA sequence variation and phylogeography of Neotropic pumas (*Puma concolor*). Mitochondrial DNA, 25, 304–312. 23789770 10.3109/19401736.2013.800486

[ece39067-bib-0004] Carbone, C. , Mace, G. M. , Roberts, S. C. , & Macdonald, D. W. (1999). Energetic constraints on the diet of terrestrial carnivores. Nature, 402, 286–288. 10580498 10.1038/46266

[ece39067-bib-0005] Chakrabarti, S. , Jhala, Y. V. , Dutta, S. , Qureshi, Q. , Kadivar, R. F. , & Rana, V. J. (2016). Adding constraints to predation through allometric relation of scats to consumption. Journal of Animal Ecology, 85, 660–670. 26931378 10.1111/1365-2656.12508

[ece39067-bib-0006] Christiansen, P. (2006). Sabertooth characters in the clouded leopard (*Neofelis nebulosa* Griffiths 1821). Journal of Morphology, 267, 1186–1198. 16845677 10.1002/jmor.10468

[ece39067-bib-0007] Clements, H. S. , Tambling, C. J. , Hayward, M. H. , & Kerley, G. I. H. (2014). An objective approach to determining the weight ranges of prey preferred by and accessible to the five large African carnivores. PLoS One, 9, e101054. 24988433 10.1371/journal.pone.0101054 PMC4079238

[ece39067-bib-0008] Davidson, P. (1998). A wildlife and habitat survey of Nam Et and Phou Louey national biodiversity conservation areas, Houaphanh province. Wildlife Conservation Society (WCS) and Centre for Protected Areas and Watershed Management (CPAWM).

[ece39067-bib-0009] D'Cruze, N. , & Macdonald, D. W. (2015). Clouded in mystery: the global trade in clouded leopards. Biodiversity and Conservation, 24, 3505–3526.

[ece39067-bib-0010] Foster, V. C. , Sarmento, P. , Sollmann, R. , Tôrres, N. , Jácomo, A. T. A. , Negrões, N. , Fonseca, C. , & Silveira, L. (2013). Jaguar and puma activity patterns and predator‐prey interactions in four Brazilian biomes. Biotropica, 45, 373–379.

[ece39067-bib-0011] Francis, C. M. (2008). A guide to the mammals of Southeast Asia. Princeton University Press.

[ece39067-bib-0012] Goodrich, J. , Lynam, A. , Miquelle, D. , Wibisono, H. , Kawanishi, K. , Pattanavibool, A. , Htun, S. , Tempa, T. , Karki, J. , Jhala, Y. , & Karanth, U. (2015). *Panthera tigris*. The IUCN Red List of Threatened Species, 2015, e.T15955A50659951. www.iucnredlist.org

[ece39067-bib-0013] Gotelli, N. J. , & Colwell, R. K. (2001). Quantifying biodiversity: Procedures and pitfalls in the measurement and comparison of species richness. Ecology Letters, 4, 379–391.

[ece39067-bib-0014] Grassman, L. I., Jr. , Tewes, M. E. , Silvy, N. J. , & Kreetiyutanont, K. (2005). Ecology of three sympatric felids in a mixed evergreen forest in north‐central Thailand. Journal of Mammalogy, 86, 29–38.

[ece39067-bib-0015] Gray, T. , Borah, J. , Coudrat, C. N. Z. , Ghimirey, Y. , Giordano, A. , Greenspan, E. , Petersen, W. , Rostro‐García, S. , Shariff, M. , & Wai‐Ming, W. (2021). *Neofelis nebulosa*. The IUCN Red List of Threatened Species, 2021, e.T14519A198843258. www.iucnredlist.org

[ece39067-bib-0016] Harmsen, B. J. , Foster, R. J. , Gutierrez, S. M. , Marin, S. Y. , & Doncaster, C. P. (2010). Scrape‐marking behavior of jaguars (*Panthera onca*) and pumas (*Puma concolor*). Journal of Mammalogy, 91, 1225–1234.

[ece39067-bib-0017] Hayward, M. W. , Jedrzejewski, W. , & Jedrzejewska, B. (2012). Prey preferences of the tiger *Panthera tigris* . Journal of Zoology, 286, 221–231.

[ece39067-bib-0018] Hayward, M. W. , O'Brien, J. , & Kerley, G. I. H. (2007). Carrying capacity of large African predators: Predictions and tests. Biological Conservation, 139, 219–229.

[ece39067-bib-0019] Jacobs, J. (1974). Quantitative measurement of food selection: A modification of the forage ratio and Ivlev's electivity index. Oecologia, 14, 413–417. 28308662 10.1007/BF00384581

[ece39067-bib-0020] Jaimes, R. P. , Caceres‐Martínez, C. H. , Acevedo, A. A. , Arias‐Alzate, A. , & González‐Maya, J. F. (2018). Food habits of puma (*Puma concolor*) in the Andean areas of Tamá National Natural Park and its buffer zone, Colombia. Therya, 9, 201–208.

[ece39067-bib-0021] Johnson, A. , Goodrich, J. , Hansel, T. , Rasphone, A. , Saypanya, S. , Vongkhamheng, C. , Venevongphet , & Strindberg, S. (2016). To protect or neglect? Design, monitoring, and evaluation of a law enforcement strategy to recover small populations of wild tigers and their prey. Biological Conservervation, 202, 99–109.

[ece39067-bib-0022] Johnson, A. , Vongkhamheng, C. , Hedemark, M. , & Saithongdam, T. (2006). Effects of human‐carnivore conflict on tiger (*Panthera tigris*) and prey populations in Lao PDR. Animal Conservation, 9, 421–430.

[ece39067-bib-0023] Kamler, J. F. , Inthapanya, X. , Rasphone, A. , Bousa, A. , Vongkhamheng, C. , Johnson, A. , & Macdonald, D. W. (2020). Diet, prey selection, and activity of Asian golden cats and leopard cats in northern Laos. Journal of Mammalogy, 101, 1267–1278.

[ece39067-bib-0024] Kamler, J. F. , Johnson, A. , Vongkhamheng, C. , & Bousa, A. (2012). The diet, prey selection, and activity of dholes (*Cuon alpinus*) in northern Laos. Journal of Mammalogy, 93, 627–633.

[ece39067-bib-0025] Kamler, J. F. , Thatdokkham, K. , Rostro‐García, S. , Bousa, A. , Caragiulo, A. , Crouthers, R. , In, V. , Pay, C. , Pin, C. , Prum, S. , Vongchamheng, C. , Johnson, A. , & Macdonald, D. W. (2020). Diet and prey selection of dholes in evergreen and deciduous forests of Southeast Asia. Journal of Wildlife Management, 84, 1396–1405.

[ece39067-bib-0026] Karanth, K. U. , Nichols, J. D. , Kumar, N. S. , Link, W. A. , & Hines, J. E. (2004). Tigers and their prey: Predicting carnivore densities from prey abundance. Proceedings of the National Academy of Sciences of the United States of America, 101, 4854–4858. 15041746 10.1073/pnas.0306210101 PMC387338

[ece39067-bib-0027] Karanth, K. U. , & Sunquist, M. E. (1995). Prey selection by tiger, leopard and dhole in tropical forests. Journal of Animal Ecology, 64, 439–450.

[ece39067-bib-0028] Kawanishi, K. , & Sunquist, M. E. (2004). Conservation status of tigers in a primary rainforest of Malaysia. Biological Conservation, 120, 329–344.

[ece39067-bib-0029] Klare, U. , Kamler, J. F. , & Macdonald, D. W. (2011). A comparison and critique of different scat‐analysis methods for determining carnivore diets. Mammal Review, 41, 294–312.

[ece39067-bib-0030] Klare, U. , Kamler, J. F. , Stenkewitz, U. , & Macdonald, D. W. (2010). Diet, prey selection, and predation impact of black‐backed jackals in South Africa. Journal of Wildlife Management, 74, 1030–1042.

[ece39067-bib-0031] Krebs, C. J. (1989). Ecological methodology. Harper and Row.

[ece39067-bib-0032] Lam, W. Y. , Hedges, L. , & Clements, G. R. (2014). First record of a clouded leopard predating on a binturong. Cat News, 60, 33.

[ece39067-bib-0033] Laurenzi, A. , Bodino, N. , & Mori, E. (2016). Much ado about nothing: Assessing the impact of a problematic rodent on agricultural and native trees. Mammal Research, 61, 65–72.

[ece39067-bib-0034] MacCracken, J. G. , & Hansen, R. M. (1987). Coyote feeding strategies in southeastern Idaho: optimal foraging by an opportunistic predator? Journal of Wildlife Management, 51, 278–285.

[ece39067-bib-0035] Macdonald, D. W. , Bothwell, H. M. , Kaszta, Ż. , Ash, E. , Bolongon, G. , Burnham, D. , Can, Ö. E. , Campos‐Arceiz, A. , Channa, P. , Clements, G. R. , Hearn, A. J. , Hedges, L. , Htun, S. , Kamler, J. F. , Kawanishi, K. , Macdonald, E. A. , Mohamad, S. W. , Moore, J. , Naing, H. , … Cushman, S. A. (2019). Multi‐scale habitat modelling identifies spatial conservation priorities for mainland clouded leopards (*Neofelis nebulosa*). Diversity and Distributions, 25, 1639–1654.

[ece39067-bib-0036] Matsuda, I. , Tuuga, A. , & Higashi, S. (2008). Clouded leopard (*Neofelis diardi*) predation on proboscis monkeys (*Nasalis larvatus*) in Sabah, Malaysia. Primates, 49, 227–231. 18484152 10.1007/s10329-008-0085-2

[ece39067-bib-0037] Miller, C. S. , Hebblewhite, M. , Petrunenko, Y. K. , Seryodkin, I. V. , Goodrich, J. M. , & Miquelle, D. G. (2014). Amur tiger (*Panthera tigris altaica*) energetic requirements: Implications for conserving wild tigers. Biological Conservation, 170, 120–129.

[ece39067-bib-0038] Morino, L. (2010). Clouded leopard predation on a wild juvenile siamang. Folia Primatologica, 81, 362–368. 10.1159/000324303 21454986

[ece39067-bib-0039] Nagy‐Reis, M. B. , Iwakami, V. H. S. , Esevo, C. A. , & Setz, E. Z. F. (2019). Temporal and dietary segregation in a neotropical small‐felid assemblage and its relation to prey activity. Mammalian Biology, 95, 1–8.

[ece39067-bib-0040] Naing, H. , Htun, S. , Kamler, J. F. , Burnham, D. , & Macdonald, D. W. (2019). Large carnivores as potential predators of sun bears. Ursus, 30, e4.

[ece39067-bib-0041] Nowak, R. M. (1999). Walker's mammals of the world. The Johns Hopkins University Press.

[ece39067-bib-0042] Nowell, K. , & Jackson, P. (1996). Wild cats. Status survey and conservation action plan. IUCN/SSC Cat Specialist Group. International Union for the Conservation of Nature.

[ece39067-bib-0043] O'Kelly, H. J. , Evans, T. D. , Stokes, E. J. , Clements, T. J. , Dara, A. , Gately, M. M. , Menghor, N. , Pollard, E. H. B. , Soriyun, M. , & Walston, J. (2012). Identifying conservation successes, failures and future opportunities; assessing recovery potential of wild ungulates and tigers in eastern Cambodia. PLoS One, 7, e40482. 23077476 10.1371/journal.pone.0040482 PMC3471919

[ece39067-bib-0044] Oksanen, J. , Blanchet, F. G. , Friendly, M. , Kindt, R. , Legendre, P. , McGlinn, D. , Minchin, P. R. , O'Hara, R. B. , Simpson, G. L. , Solymos, P. , Stevens, M. H. H. , Szoecs, E. , & Wagner, H. (2019). Package ‘vegan.’ https://github.com/vegandevs/vegan

[ece39067-bib-0045] Pakpien, S. , Simcharoen, A. , Duangchantrasiri, S. , Chimchome, V. , Pongpattanurak, N. , & Smith, J. L. D. (2017). Ecological correlates at kill sites influence tiger (*Panthera tigris*) hunting success in Huai Kha Khaeng Wildlife Sanctuary, Thailand. Tropical Conservation Science, 10, 1–7.

[ece39067-bib-0046] Phan, T. D. , Nijhawan, S. , Li, S. , & Xiao, L. (2020). *Capricornis sumatraensis*. The IUCN Red List of Threatened Species, 2020, e.T162916735A162916910. www.iucnredlist.org

[ece39067-bib-0047] Pocock, R. I. (1939). The fauna of British India. Mammalia, I. Primates and carnivore. Taylor and Francis.

[ece39067-bib-0048] Prommakul, P. (2003). Habitat utilization and prey of the tiger (*Panthera tigris* [Linnaeus]) in the eastern Thung Yai Naresaun Wildlife Sanctuary. Journal of Wildlife in Thailand, 11, 1–12 [in Thai with English Summary.].

[ece39067-bib-0049] R Core Team . (2021). R: A language and environment for statistical computing. R Foundation for Statistical Computing.

[ece39067-bib-0050] Rabinowitz, A. (1989). The density and behavior of large cats in a dry tropical forest mosaic in Huai Kha Khaeng Wildlife Sanctuary, Thailand. Natural History Bulletin of the Siam Society, 37, 235–251.

[ece39067-bib-0051] Rabinowitz, A. , Andau, P. , & Chai, P. P. K. (1987). The clouded leopard in Malaysian Borneo. Oryx, 21, 107–111.

[ece39067-bib-0052] Rasphone, A. , Kamler, J. F. , & Macdonald, D. W. (2020). Temporal partitioning by felids, dholes, and their potential prey in northern Laos. Mammal Research, 65, 679–689.

[ece39067-bib-0053] Rasphone, A. , Kamler, J. F. , Tobler, M. , & Macdonald, D. W. (2021). Density trends of wild felids in northern Laos. Biodiversity and Conservation, 30, 1881–1897.

[ece39067-bib-0054] Rasphone, A. , Kéry, M. , Kamler, J. F. , & Macdonald, D. W. (2019). Documenting the demise of tiger and leopard, and the status of other carnivores and prey, in Lao PDR's most prized protected area: Nam Et‐Phou Louey. Global Ecology and Conservation, 20, e00766.

[ece39067-bib-0055] Rodgers, T. W. , Giacalone, J. , Heske, E. J. , Pawlikowski, N. C. , & Schooley, R. L. (2015). Communal latrines act as potentially important communication centers in ocelots *Leopardus pardalis* . Mammalian Biology, 80, 380–384.

[ece39067-bib-0056] Rostro‐García, S. , Kamler, J. F. , & Hunter, L. T. B. (2015). To kill, stay or flee: The effects of lions and landscape factors on habitat and kill site selection of cheetahs in South Africa. PLoS One, 10, e0117743. 25693067 10.1371/journal.pone.0117743 PMC4333767

[ece39067-bib-0057] Sandom, C. J. , Faurby, S. , Svenning, J.‐C. , Burnham, D. , Dickman, A. J. , Hinks, A. E. , Macdonald, E. A. , Ripple, W. J. , Williams, J. , & Macdonald, D. W. (2018). Learning from the past to prepare for the future: Felids face continued threat from declining prey. Ecography, 41, 140–152.

[ece39067-bib-0058] Sandom, C. J. , Williams, J. , Burnham, D. , Dickman, A. J. , Hinks, A. E. , Macdonald, E. A. , & Macdonald, D. W. (2017). Deconstructed cat communities: Quantifying the threat to felids from prey depletion. Diversity and Distributions, 23, 667–679.

[ece39067-bib-0059] Schaller, G. B. (1967). The deer and the tiger: A study of wildlife in India. University of Chicago Press.

[ece39067-bib-0060] Seryodkin, I. V. , Miquelle, D. G. , Goodrich, J. M. , Kostyria, A. V. , & Petrunenko, Y. K. (2018). Interspecific relationships between the Amur tiger (*Panthera tigris altaica*) and brown (*Ursus arctos*) and Asiatic black (*Ursus thibetanus*) bears. Biology Bulletin, 45, 853–864.

[ece39067-bib-0061] Simcharoen, A. , Simcharoen, S. , Duangchantrasiri, S. , Bump, J. , & Smith, J. L. D. (2018). Tiger and leopard diets in western Thailand: Evidence for overlap and potential consequences. Food Webs, 15, e00085.

[ece39067-bib-0062] Sunderland‐Groves, J. L. , Tandang, M. V. , Patispathika, F. H. , Marzec, A. , Knox, A. , Nurcahyo, A. , Husson, S. J. , & Sihite, J. (2021). Suspected Sunda clouded leopard (*Neofelis diardi*) predation attempts on two reintroduced Bornean orangutans (*Pongo pygmeaus wurmbii*) in Bukit Batikap Protection Forest, Central Kalimantan, Indonesia. Primates, 62, 41–49. 32623603 10.1007/s10329-020-00842-1

[ece39067-bib-0063] Sunquist, M. , Karanth, K. U. , & Sunquist, F. (1991). Ecology, behaviour and resilience of the tiger and its conservation needs. In J. Seidensticker , S. Christie , & P. Jackson (Eds.), Riding the tiger: tiger conservation in human‐dominated landscapes (pp. 5–18). Cambridge University Press.

[ece39067-bib-0064] Ten, D. C. Y. , Jani, R. , Hashim, N. H. , Saaban, S. , Hashim, A. K. A. , & Abdullah, M. T. (2021). *Panthera tigris jacksoni* population crash and impending extinction due to environmental perturbation and human‐wildlife conflict. Animals, 11, 1032. 33917373 10.3390/ani11041032 PMC8067357

[ece39067-bib-0065] Vongkhamheng, C. (2011). Abundance and distribution of tiger and prey in montane tropical forest in northern Lao People Democratic Republic. Dissertation, University of Florida, Gainesville, Florida, USA.

[ece39067-bib-0066] Vongkhamheng, C. , Johnson, A. , & Sunquist, M. E. (2013). A baseline survey of ungulate abundance and distribution in northern Lao: Implications for conservation. Oryx, 47, 544–552.

[ece39067-bib-0067] Yang, H. , Zhao, X. , Han, B. , Wang, T. , Mou, P. , Ge, J. , & Feng, L. (2018). Spatiotemporal patterns of Amur leopards in northeast China: Influence of tigers, prey, and humans. Mammalian Biology, 92, 120–128.

